# Tracking of sport and exercise types from midlife to old age: a 20-year cohort study of British men

**DOI:** 10.1186/s11556-018-0205-y

**Published:** 2018-12-07

**Authors:** Daniel Aggio, Olia Papacosta, Lucy T. Lennon, Sarah Ash, Peter H. Whincup, S. Goya Wannamethee, Barbara J. Jefferis

**Affiliations:** 1 UCL Department of Primary Care & Population Health, UCL Medical School, Royal Free Campus, Rowland Hill Street, London, NW3 2PF UK; 2 UCL Physical Activity Research Group, London, UK; 3 Population Health Research Institute, St George’s University of London, Cranmer Terrace, London, SW17 0RE UK

**Keywords:** Aging, Physical activity, Longitudinal, Stability

## Abstract

**Background:**

Previous physical activity (PA) tracking studies have examined the stability of overall PA and/or PA types, but few have investigated how specific types of sport/exercise track over the life course. The aim of this study was to determine how specific sports/exercises in midlife track and predict future sport/exercise and PA in men transitioning to old age.

**Methods:**

Seven thousand seven hundred thirty-five men (aged 40–59 years) recruited in 1978–80 were followed up after 12, 16 and 20 years. At each wave men self-reported participation in sport/exercise. Frequent sport/exercise participants (> 1/month) reported the types of sport/exercise they engaged in. Men also reported total PA, health status, lifestyle behaviours and socio-demographic characteristics. Stability of each sport/exercise was assessed using kappa statistics and intraclass correlation coefficients. Logistic regression estimated the odds of participating in sport/exercise and being active at 20-year follow up according to specific types of sport/exercise in midlife.

**Results:**

Three thousand three hundred eighty-four men with complete data at all waves were included in analyses. Tracking of specific sports/exercises ranged from fair to substantial, with golf being the most common and most stable. Bowls was the most frequently adopted. Odds of participating in sport/exercise and being active in old age varied according to sport/exercise types in midlife. Golf and bowls in midlife were the strongest predictors of sport/exercise participation in old age. Golf, cricket and running/jogging in midlife were among the strongest predictors of being active in old age. Compared to participating in just one sport/exercise in midlife, sampling multiple sports/exercises was more strongly associated with sport/exercise participation and being active in old age.

**Conclusion:**

The stability of sport/exercise participation from midlife to old age varies by type. Specific sports/exercises in midlife may be more likely to predict future PA than others. However, participating in a range of sports/exercises may be optimal for preserving PA into old age.

**Electronic supplementary material:**

The online version of this article (10.1186/s11556-018-0205-y) contains supplementary material, which is available to authorized users.

## Background

Sport and exercise is an important component of physical activity (PA) [1] and contributes considerably to the physiological benefits related to PA [2]. Moreover, participation in sport, and particularly team sports, is associated with better psychological health, largely due to the social aspect of engagement [3]. Furthermore, several prospective cohort studies have shown that sport participation more strongly predicts PA later in life than other types of activity [4, 5]. Thus, increasing sport/exercise participation remains a highly important strategy for promoting lifelong PA.

While we know that sport/exercise participation is a stronger predictor of future activity levels than other types of PA, such as active play, walking and active travel [4–6], very few prospective studies have been able to track participation in specific types of sport/exercise. Given that the health benefits of sport might be specific to type, [2, 7] it is important to understand the stability of specific sports and exercises during the transition to old age, a period when participation is potentially modifiable [8]. Cross-sectional evidence suggests that participation in particular types of sport/exercise varies at different life stages [9]. Temporal trends in sport/exercise participation have also been shown to vary according to type [10]. For example, notable increases in gym and fitness club attendance have been observed over recent decades in UK adults aged over 45 [10]. However, it remains unknown whether attendance at gyms/fitness classes or other types of sport/exercise are likely to be maintained into old age. It may be possible that certain sports/exercises, or multiple sports/exercises [11], are fundamental for establishing a lifelong habit for sport and PA. Identifying the tracking of specific sports/exercises could help inform intervention strategies to promote uptake and maintenance of sport/exercise participation into old age.

We hypothesised that the stability and predictability of sport/exercise participation in old age would be specific to type. The aim of this study was to estimate the tracking of specific sports and exercises in men making the transition from midlife to old age and the predictability of sport/exercise participation and PA in old age from participation in specific sports/exercises in midlife.

## Methods

### Participants

The British Regional Heart Study (BRHS) is an ongoing prospective cohort study following up 7735 men (response rate = 78%) recruited from primary care practices in 24 towns in Great Britain [12]. Men attended a physical examination at baseline in 1978–80 when they were aged 40–59 years and at the 20-year follow up. Men were also followed up after 12 and 16 years via postal questionnaires. Those who did not respond to the questionnaires or examination invites were sent a reminder in the post. Response rates for surviving cohort members were 91% (*n* = 5925), 88% (*n* = 5263) and 77% (*n* = 4252) at 12-, 16- and 20-year follow ups, respectively. Men completed a lifestyle and medical history questionnaire at each follow up. Participants provided informed written consent to the investigation in accordance with the Declaration of Helsinki. Ethical approval was obtained from the National Research Ethics Service Committee London.

### Measures

#### Self-reported physical activity & sport and exercise

At baseline and subsequent follow ups, men reported their usual PA levels including time spent walking and participating in recreational activities (such as recreational walking, gardening, chores and do-it-yourself activities) and how often they participated in sport/exercise. Responses were scored based on the intensity and frequency of the activity [13, 14]. For example, making no journeys by foot was scored as 0 and > 90 min/weekday was scored as 5. Scores were also heavily weighted for vigorous activities. For example, playing sport 4–7 times a month was given a score of 8. Scores for each PA type were summed to generate a total PA index. The original scoring system has been described in detail elsewhere [15]. The index was then used to group men into 6 categories (0–5): inactive, occasional, light, moderate, moderately vigorous or vigorous. This 6-point score previously been validated against resting heart rate [15] and device-measured PA [16]. For the purposes of this study the 6 categories of the index were grouped into active or inactive (inactive and occasional groups were classified as inactive). For sport/exercise participation, men were asked “Do you take active physical exercise such as running, swimming, golf, tennis, squash, jogging, bowls, cycling etc?” Response options were ‘no’, ‘occasionally (less than once a month)’ and ‘frequently (once a month or more)’. Men who reported ‘no’ or ‘occasionally’ were classified as ‘non-sport participants’ and men reporting ‘frequently’ as ‘sport participants’. Men reporting frequent sport/exercise participation were also asked to state the type of sport/exercise they engaged in. Men reporting activities not deemed to be sport or exercise (e.g. snooker) were reclassified as non-sport participants. Cycling participation combined all forms, including men who reported frequent cycling for transport purposes. Men reporting walking for transport purposes were not included as ‘walking/hiking’ participants. As participation in some sports/exercises was low, similar sports were grouped together. For example, bowls, curling and skittles were combined to form ‘bowling games’. A full list of sports/exercises that were combined can be viewed in Additional file 1. Sports/exercises that were extremely rare (≤0.5%) were also combined into an ‘Other’ category. Men were also classified according to the number of sports/exercises they engaged in (none, one, two, three or more).

### Covariates

At baseline, men reported their age, occupational class (manual vs. non-manual) and smoking status (current/recent ex-smokers vs. never smokers). Body Mass Index (BMI, overweight/obese [≥25.0 Kg/m^2^] vs. healthy weight [25.0 Kg/m^2^]) was calculated from heights and weights measured by nurses. Season (summer/winter) was derived from the questionnaire completion date at baseline and 20-year follow up.

### Statistical analysis

Descriptive statistics were used to report the baseline characteristics of the sample and the proportion of men participating in each type of sport/exercise at each wave. The proportion of men who sustained and changed their participation (adopters and drop outs) in sport/exercise and sport/exercise types between baseline and the 20-year follow up was also estimated. Cohen’s kappa was used to assess the observed agreement compared with the expected agreement between baseline and subsequent waves. We followed suggestions by Munoz and Bangdiwala for interpretation of K coefficients: < 0.00 indicates poor agreement, 0.00–0.20 fair agreement, 0.21–0.45 moderate agreement, 0.46–0.75 substantial agreement and 0.76–1.0 indicates near perfect agreement [17]. Random effects logit models were also used to calculate intraclass correlation coefficients (ICCs) [18], an indicator of tracking, using the binary measures of participation across all assessments whilst also controlling for baseline variables including age, entered as a continuous variable, BMI, social class, smoking status (categorical) and season of questionnaire completion at baseline and 20-year follow up. Finally, we used logistic regression to estimate the odds ratio for participating in sport/exercise and being physically active at the 20-year follow up according to participation in specific types of sport/exercise at baseline. In a separate model, we included the number of sports/exercises participants engaged in (0, 1, 2, > 3) as an exposure variable to examine whether participation in a higher number of sports/exercise in midlife was associated with increased odds of being active and participating in sport/exercise at the 20-year follow up. Initial models were adjusted for age (model 1) and then for BMI, social class, smoking status (categorical) and season of questionnaire completion at the baseline and 20-year follow up (model 2). Model 3 included all sports/exercise types at baseline simultaneously in the model whilst also including adjustments made in model 2. Model 4 included adjustments made in model 2 and total PA score at baseline.

### Additional analyses

To understand whether the tracking of specific sports/exercises differed according to occupational class, participation and change in participation (adopters and drop outs) was also estimated for men from manual and non-manual occupations separately. In addition, further investigations were conducted to establish whether men who dropout of specific sports/exercises are likely to take up other sports/exercises. As the cycling participation variable combined leisure and transport cycling, we also estimated the odds of sport/exercise participation and PA at 20-year follow up according to leisure cycling only.

## Results

Out of 7735 men originally invited at the baseline survey, 4118 men with missing sport/exercise participation data at any of the four waves were excluded. An additional 233 men were excluded due to missing covariate data, leaving a final sample of 3384. At baseline, compared to men in the final sample, men who were excluded from the analysis (*n* = 4351) were older (51.4 vs. 48.8 years, *p* < 0.05), less active (≥light activity, 56.6% vs. 65.0%, *p* < 0.05), participated in less sport (frequent participation, 26.7% vs. 38.2%, *p* < 0.05), were more likely to have a manual occupation (67.6% vs. 51.0%, *p* < 0.05), smoke (49.6% vs. 30.5%, *p* < 0.05) and be overweight or obese (55.5% vs. 52.6%, *p* < 0.05).

Sample characteristics are displayed in Table 1. The mean age of the men was 48.8 (SD = 5.5, range 38–60) and 68.7 (SD = 5.5, range 58–81) years at baseline and 20-year follow up, respectively. Around two thirds of men were classified as being active and around a third as frequent sport participants at baseline, which remained fairly constant over the 20 years of follow up. A total of 523 men who did not participate in sport/exercise at baseline adopted a sport/exercise by the 20-year follow up and 536 men who participated in sport/exercise at baseline subsequently dropped out by the 20-year follow up. Table 2 presents the proportion of men who were participating in specific sports/exercises at each wave and the proportion who sustained or changed their participation from baseline to the 20-year follow up. Golf was the most common with around 12% reporting participation at all waves. Racquet sports were the next most common at baseline, but a large decrease was observed between baseline (9.3%) and the 20-year follow up (2.2%). Cycling also decreased from 7.3 to 6.1%, surface water sports from 2.1 to 0.4%, football from 0.7 to 0.1%, rugby from 0.2 to 0.0%, running/jogging from 2.3 to 1.1%, cricket from 1.6 to 0.1% and other sports from 3.5% at baseline to 1.9% at 20-year follow up. Swimming increased from 6.9 to 9.2%, dancing from 1.2 to 5.1%, bowls from 2.2 to 9.5%, aerobics/fitness classes from 0.2 to 1.5% and gym/muscle strengthening from 1.1% at baseline to 1.9% at the 20-year follow up. Bowls (8.0%), walking (7.5%) and swimming (7.4%) were the most frequently adopted sports between baseline and 20-year follow up, followed by golf (4.8%), cycling (4.7%) and dancing (4.5%). Racquet sports had the highest proportion of dropouts (7.9%), followed by cycling (5.9%), swimming (5.1%) and golf (4.5%). Out of these sports, those who dropped out of racquet sports were the most likely to take part in another sport/exercise at the 20-year follow up (62%, *n* = 164), followed by golf dropouts (39%, *n* = 59), swimming dropouts (38%, *n* = 65) and cycling dropouts (28%, *n* = 55).

**Table 1:** Sample characteristics, sport and exercise participation and physical activity levels at baseline, 12-, 16- and 20-year follow up, *n* = 3384

Characteristic	Baseline	12 year	16 year	20 year
Age (years, mean ± SD)	48.8 ± 5.5	62.4 ± 5.5	66.4 ± 5.5	68.7 ± 5.5
Overweight/Obese (%, n)	52.6 (1780)			
Current smoker (%, n)	30.5 (1033)			
Manual Occupation (%, n)	51.0 (1727)			
Physically active^a^ (%, n)	65.0 (2175)	70.0 (2297)	62.6 (2037)	65.7 (2180)
Frequent sport/exer. participants^b^ (%, n)	38.2 (1292)	36.8 (1244)	37.1 (1254)	37.8 (1279)
Sport/exercise change Q1 to Q20				
Persistent non-participants	46.4 (1569)			
Adopters	15.5 (523)			
Dropouts	15.8 (536)			
Persistent participants	22.3 (756)			

Data presented are for participants who reported their sport/exercise participation at all four time points (n = 3384)

*SD*, standard deviation

^a^Physically active was classified as at least light activity in a total physical activity index of walking, recreational activity and sport. The sample size was lower for total physical activity than for sport as men with incomplete data from the other domains were not assigned a score. Numbers of men with a valid physical activity score were 3347 at baseline, 3283 at the 12-year follow up, 3255 at the 16-year follow up and 3318 at the 20-year follow up

^b^Frequent sport and exercise participation (once a month or more)

**Table 2:** Participation and change in sport and exercise participation over 20 years of follow up, n = 3384 men

					Change between baseline and 20-year follow up			
Sport/exercise type	Baseline	12 year	16 year	20 year	Participating at both	Adopters	Drop outs	Not participating at both
	% (n)							
Golf	11.4 (387)	11.8 (400)	11.7 (397)	11.7 (396)	6.9 (234)	4.8 (162)	4.5 (153)	83.8 (2835)
Bowling	2.2 (74)	8.4 (285)	9.5 (322)	9.5 (321)	1.5 (51)	8.0 (270)	0.7 (23)	89.8 (3040)
Dancing	1.2 (42)	1.7 (59)	2.0 (68)	5.1 (172)	0.6 (20)	4.5 (152)	0.7 (22)	94.3 (3190)
Racquet sports	9.3 (314)	3.6 (123)	2.2 (74)	2.2 (73)	1.4 (48)	0.7 (25)	7.9 (266)	90.0 (3045)
Swimming	6.9 (235)	9.6 (324)	9.0 (305)	9.2 (312)	1.8 (62)	7.4 (250)	5.1 (173)	85.7 (2899)
Cycling (any purpose)	7.3 (246)	8.9 (300)	8.2 (276)	6.1 (206)	1.4 (46)	4.7 (160)	5.9 (200)	88.0 (2978)
Surface water sports	2.1 (71)	1.0 (32)	0.7 (25)	0.4 (15)	0.2 (6)	0.3 (9)	1.9 (65)	97.6 (3304)
Aerobics/fitness training	0.2 (5)	0.7 (24)	0.8 (27)	1.5 (52)	0.0 (1)	1.5 (51)	0.1 (4)	98.4 (3328)
Gym/muscle strengthening	1.1 (38)	1.6 (55)	2.2 (75)	1.9 (65)	0.1 (4)	1.8 (61)	1.0 (34)	97.1 (3285)
Football	0.7 (25)	0.1 (4)	0.2 (6)	0.1 (2)	–	0.1 (2)	0.7 (25)	99.2 (3357)
Rugby	0.2 (6)	0.0 (0)	0.0 (0)	0.0 (0)	–	–	0.2 (6)	99.8 (3378)
Running/jogging	2.3 (77)	1.2 (42)	1.0 (35)	1.1 (36)	0.4 (14)	0.7 (22)	1.9 (63)	97.1 (3285)
Cricket	1.6 (54)	0.3 (9)	0.2 (6)	0.1 (4)	0.1 (3)	0.0 (1)	1.5 (51)	98.4 (3329)
Walking/hiking	1.4 (47)	6.5 (219)	7.4 (251)	8.0 (272)	0.6 (19)	7.5 (253)	0.8 (28)	91.1 (3084)
Other	3.3 (113)	2.2 (74)	1.9 (65)	1.7 (58)	0.4 (12)	1.4 (46)	3.0 (101)	95.3 (3225)

Golf was by far the most common sport/exercise to be sustained with 6.9% participating at baseline and at the 20-year follow up, followed by swimming with 1.8% participating at both time points. Additional analyses stratifying by occupational class showed that some sports were more often played by men from non-manual occupational classes. For example, golf was played by 14.2% of men from non-manual classes compared to 8.8% of men from manual classes at baseline (see Additional files 2 and 3). Racquet sports, swimming, gym/muscle strengthening and running/jogging were also more prevalent among those from non-manual classes. Golf (6.5% vs. 3.1%), racquet sports (11.9% vs. 4.0%), walking/hiking (9.5% vs. 5.6%) and swimming (8.9% vs. 6.0%) were more frequently adopted in men from non-manual occupations compared to men from manual occupations, but adoption was comparable for bowls (8.5% vs 7.5%), dancing (4.0% vs 4.9%) and cycling (4.8% vs. 4.6%).

Kappa statistics were highest for golf ranging from 0.55 to 0.60 indicating substantial levels of agreement between baseline and subsequent waves (see Table 3). ICCs also demonstrated high levels of tracking for golf that were only marginally attenuated after adjusting for age, BMI, social class, smoking status and season. For bowling, dancing, racquet sports, swimming, cycling, surface water sports and running/jogging kappa statistics demonstrated fair to moderate levels of agreement; however, in random effects models which utilise data across all available waves, ICCs suggested high levels of tracking for bowling. The majority of the remaining sports/exercises demonstrated lower levels of tracking. In random effects models including men with  only one measure of sport/exercise participation, ICCs were marginally weakened (data not shown). However, the overall conclusions remained the same.

**Table 3:** Stability (Kappa statistics and intra class correlation coefficients [ICC]) of sport and exercise type participation across 4 waves^a^, *n* = 3384 men

				Random Effects Models	
Sport/exercise type	Wave 1 to 2	Wave 1 to 3	Wave 1 to 4	Univariate	Multivariate^b^
	Kappa	Kappa	Kappa	ICC (95% CI)	ICC (95% CI)
Golf	0.60	0.57	0.55	0.93 (0.92, 0.93)	0.89 (0.88, 0.90)
Bowling	0.28	0.22	0.23	0.85 (0.84, 0.86)	0.85 (0.84, 0.86)
Dancing	0.29	0.24	0.17	0.71 (0.67, 0.74)	0.80 (0.76, 0.83)
Racquet sports	0.34	0.23	0.22	0.72 (0.69, 0.75)	0.72 (0.66, 0.77)
Swimming	0.17	0.15	0.16	0.65 (0.62, 0.69)	0.64 (0.61, 0.68)
Cycling (any purpose)	0.30	0.23	0.15	0.65 (0.61, 0.68)	0.64 (0.60, 0.68)
Surface water sports	0.34	0.16	0.13	0.79 (0.75, 0.82)	0.70 (0.65, 0.74)
Aerobics/fitness training	0.07	0.06	0.03	0.67 (0.60, 0.74)	0.63 (0.54, 0.72)
Gym/muscle strengthening	0.12	0.13	0.06	0.74 (0.68, 0.80)	0.70 (0.62, 0.78)
Football	0.07	0.00	0.00	0.67 (0.55, 0.77)	0.66 (0.52, 0.78)
Rugby	0.00	0.00	0.00	^c^	^c^
Running/jogging	0.26	0.24	0.24	0.78 (0.74, 0.81)	0.77 (0.65, 0.86)
Cricket	0.19	0.10	0.10	0.73 (0.67, 0.79)	0.71 (0.60, 0.79)
Walking/hiking	0.08	0.09	0.10	0.58 (0.53, 0.63)	0.56 (0.51, 0.61)
Other	0.25	0.15	0.12	0.67 (0.60, 0.74)	0.65 (0.57, 0.72)

^a^wave 1 = baseline, wave 2 = 12-year follow up, wave 3 = 16-year follow up, wave 4 = 20-year follow up

^b^adjusted for age, BMI, social class, smoking status at baseline and season

^c^Models failed to converge

Table 4 presents the odds ratios for participating in sport/exercise monthly or more compared to no or less frequent participation and for being active (in any of sport, recreational activity and walking) compared to inactive at the 20-year follow up according to participation in specific types of sport/exercise at baseline. Playing golf (OR 6.6, 95% CI 5.5, 9.0) and bowls (OR 7.7, 95% CI 4.4, 13.5) at baseline were the strongest predictors of participating in sport/exercise 20 years later after adjusting for age, BMI, social class, smoking status, season and participation in other sports. Odds of sport/exercise participation at 20-year follow up were also raised for men who participated in dance (OR 4.3, 95% CI 2.3, 8.2), racquet sports (OR 3.0, 95% CI 2.3, 4.0), running/jogging (OR 2.6, 95% CI 1.6, 4.3), other sports (OR 2.0, 95% CI 1.3, 3.0), surface water sports (OR 2.0, 95% CI 1.2, 3.4), cricket (OR 2.1, 95% CI 1.2, 3.5), walking (OR 2.1 95% CI 1.1, 3.9) and swimming (OR 1.6, 95% CI 1.2, 2.1) at baseline. Cycling and gym/muscle strengthening were only weakly associated with sport/exercise at 20-year follow up after accounting for participation in all other sports/exercises. Associations were largely attenuated after adjusting for total PA score at baseline. Only golf (OR 3.4 95% CI 2.6, 4.5), bowls (OR 3.0 95% CI 1.7, 5.3) and racquet sports (OR 1.6, 95% CI 1.2, 2.2) remained significant. Similar associations were observed for being active at the 20-year follow up. Golf (OR 4.2, 95% CI 3.0, 5.7), cricket (OR 3.0, 95% CI 1.2, 7.2) and running/jogging (OR 3.2, 95% CI 1.5, 6.8) in midlife were amongst the strongest predictors of being active 20 years later after adjusting for age, BMI, social class, smoking status, season and participation in other sports. Odds ratios of being active were also raised for dancing, bowls, racquet sports, swimming, cycling and other sports/exercises. Only golf remained significant after additionally adjusting for total PA score at baseline (OR 2.1, 95% CI 1.5, 2.9) Odds ratios for cycling were raised when only leisure cycling was considered at baseline; however, participation for leisure purposes was low at baseline and so power to detect associations was limited (data not shown). There was also a dose-response relationship between the number of sports/exercises participated in during midlife and odds of still participating in sport/exercise and being active 20 years later, with 3 or more sports/exercises proving optimal. Multiple sport participation (≥2) at baseline was most common among men who took part in swimming (53.2%), running (66.7%), racquet sports (54.4%) and cricket (66.1%), data not shown.

**Table 4:** Odds Ratios (ORs) of participating in sport and exercise or not and being active or not at 20-year follow up according to type of sport and exercise type participation at baseline, n = 3384 and 3318 men

Sport/exercise type	Participating in sport/exercise at 20-year follow up (*n* = 3384)				Physically active at 20-year follow up (*n* = 3318)			
	Model 1	Model 2	Model 3	Model 4^a^	Model 1	Model 2	Model 3	Model 4^b^
	OR (95% CI)	OR (95% CI)	OR (95% CI)	OR (95% CI)	OR (95% CI)	OR (95% CI)	OR (95% CI)	OR (95% CI)
Golf	**6.3 (4.9, 8.0)**	**6.1 (4.8, 7.8)**	**6.6 (5.1, 8.5)**	**3.4 (2.6, 4.5)**	**4.2 (3.0, 5.7)**	**4.1 (3.0, 5.6)**	**4.2 (3.0, 5.7)**	**2.1 (1.5, 2.9)**
Bowling	**5.9 (3.4, 10.1)**	**6.1 (3.5, 10.6)**	**7.7 (4.4, 13.5)**	**3.0 (1.7, 5.3)**	**2.3 (1.3, 4.1)**	**2.3 (1.3, 4.1)**	**2.5 (1.4, 4.6)**	1.0 (0.5, 1.8)
Dancing	**3.1 (1.6, 5.7)**	**3.4 (1.8, 6.4)**	**4.3 (2.3, 8.2)**	1.6 (0.9, 3.2)	**2.2 (1.0, 4.6)**	**2.3 (1.0, 4.8)**	**2.6 (1.2, 5.5)**	1.1 (0.5, 2.3)
Racquet sports	**3.7 (2.9, 4.8)**	**3.4 (2.6, 4.3)**	**3.0 (2.3, 4.0)**	**1.6 (1.2, 2.2)**	**3.0 (2.2, 4.1)**	**2.7 (2.0, 3.8)**	**2.3 (1.6, 3.2)**	1.2 (0.9, 1.7)
Swimming	**2.0 (1.5, 2.6)**	**1.8 (1.4, 2.4)**	**1.6 (1.2, 2.1)**	0.8 (0.6, 1.1)	**2.6 (1.8, 3.7)**	**2.5 (1.7, 3.5)**	**2.1 (1.5, 3.0)**	1.1 (0.8, 1.6)
Cycling (any purpose)	1.2 (0.9, 1.5)	1.2 (0.9, 1.5)	**1.3 (1.0, 1.8)**	**0.6 (0.5, 0.8)**	**1.6 (1.2, 2.1)**	**1.5 (1.1, 2.1)**	**1.6 (1.2, 2.2)**	0.8 (0.6, 1.1)
Surface water sports	**2.4 (1.5, 3.8)**	**2.1 (1.3, 3.4)**	**2.0 (1.2, 3.4)**	1.1 (0.7, 1.8)	1.5 (0.9, 2.6)	1.3 (0.8, 2.3)	1.1 (0.6, 2.0)	0.6 (0.4, 1.1)
Gym/ muscle strengthening	1.7 (0.9, 3.1)	1.4 (0.8, 2.7)	**2.1 (1.1, 4.0)**	0.5 (0.3, 1.0)	1.9 (0.9, 4.2)	1.7 (0.8, 3.7)	1.9 (0.9, 4.2)	0.5 (0.2, 1.2)
Running/jogging	**3.2 (2.0, 5.1)**	**2.9 (1.8, 4.7)**	**2.6 (1.6, 4.3)**	1.1 (0.7, 1.8)	**4.2 (2.0, 8.8)**	**3.9 (1.8, 8.1)**	**3.2 (1.5, 6.8)**	1.4 (0.6, 2.9)
Cricket	**2.3 (1.3, 3.9)**	**2.1 (1.2, 3.7)**	1.6 (0.9, 3.0)	1.2 (0.7, 2.1)	**3.8 (1.6, 9.0)**	**3.7 (1.6, 8.8)**	**3.0 (1.2, 7.2)**	2.0 (0.8, 4.8)
Walking/hiking	**2.7 (1.5, 4.8)**	**2.3 (1.2, 4.1)**	**2.1 (1.1, 3.9)**	1.4 (0.8, 2.6)	**2.6 (1.2, 5.5)**	**2.2 (1.0, 4.7)**	2.0 (0.9, 4.4)	1.3 (0.6, 2.8)
Other	**2.5 (1.7, 3.7)**	**2.5 (1.7, 3.7)**	**2.1 (1.4, 3.1)**	1.1 (0.7, 1.7)	**2.8 (1.6, 4.7)**	**2.7 (1.6, 4.6)**	**2.3 (1.3, 4.0)**	1.1 (0.7, 2.0)
**Number of sports/exercises**								
0 (*n* = 2091)	1.0	1.0	–	1.0	1.0	1.0	–	1.0
1 (*n* = 940)	**3.5 (3.0, 4.1)**	**3.3 (2.8, 3.9)**	**–**	**2.0 (1.6, 2.5)**	**2.7 (2.3, 3.2)**	**2.6 (2.2, 3.1)**	**–**	1.1 (0.9, 1.4)
2 (*n* = 276)	**5.7 (4.4, 7.5)**	**5.3 (4.1, 7.0)**	**–**	**2.8 (1.9. 3.9)**	**4.5 (3.2, 6.4)**	**4.2 (3.0, 6.1)**	**–**	1.4 (0.9, 2.1)
3+ (*n* = 77)	**15.8 (8.5, 29.5)**	**14.6 (7.8, 27.3)**	**–**	**7.0 (3.6, 13.7)**	**13.0 (4.7, 35.9)**	**12.2 (4.4, 33.5)**	**–**	**3.6 (1.2, 10.1)**

N.B. Rugby, football and aerobics were not included in these models as participation at baseline was too low to generate reliable estimates

Bold indicates statistical significance p < 0.05

Model 1 = baseline age. Model 2 = model1 + social class, BMI, smoking status at baseline and season. Model 3 = Model 2 + participation in all sport and exercises at baseline. Model 4 = Model 2 + total physical activity score at baseline

^a^Total physical activity score at baseline was missing for 37 men resulting in a final sample of 3347

^b^Total physical activity score at baseline was missing for 37 men resulting in a final sample of 3281

## Discussion

To our knowledge this is the first study to examine the long-term tracking of specific sports and exercises from midlife to old age. Previous findings from the BRHS showed that even occasional or more frequent sport/exercise in midlife more than doubled the odds of being active in old age (60 to 79 years) [5]. However, the tracking of individual sports and exercises was masked as only total sport/exercise participation was studied. In the present study, we investigated specific sport/exercise types and found that tracking levels for specific sports and exercises ranged from fair to substantial. Golf was the sport most commonly played, the most stable and the strongest predictor of sport/exercise participation and being active in old age. In addition, participating in multiple sports and exercises more strongly predicted sport/exercise participation and activity in old age than taking part in a single sport/exercise alone.

Our results also highlighted sports and exercises that were more susceptible to change during the transition to old age. Tracking of racquet sports was lower owing to a large proportion dropping out of these sports. Levels of tracking were also lower for dance but this was due to a high proportion of uptake. A high level of uptake between baseline and the 12-year follow up was also observed for bowling, but participation was stable thereafter. Swimming was one of the most common sport/exercises reported throughout follow up; however, tracking levels were lower than other sports/exercises due to both high uptake and low adherence. Golf and bowls have also been highlighted as being among the most stable sports/exercises between leaving school and late midlife in Scottish adults [19]. Our study supports these findings but in an older sample of men. Anecdotally, there may be a number of factors explaining the higher levels of tracking for golf and bowls compared to other sports/exercises. For example, the age-related health barriers to PA may be less strong for these lower intensity sport/exercises, there may be a lower risk of injury, the social benefits may be greater and increased free time as a result of retirement may increase preference for these kinds of sports/exercises. Age-related health barriers, social interaction and free time are widely reported as important factors related to PA participation in older adults [20–22]. Conversely, onset of age-related health problems may be more of a barrier for higher intensity activities such as racquet sports. While we know that the barriers to sport/exercise likely change with age [23], further investigation is required to fully understand motives for long-term maintenance and changes to participation of specific sports/exercises. Small increases in aerobic/fitness training and gym activities were also observed in our sample. This may be due to a secular shift in preference for this type of activity and increased access to leisure facilities over recent decades. Temporal increases in gym and fitness-based activities have previously been reported in UK adults over a similar period [10]. Furthermore, we found that participation in the majority of sports and exercises was more common in men from more advantaged socioeconomic backgrounds, with large disparities observed for golf, racquet sports and swimming. Providers of these activities should seek to minimise the barriers to participation in more disadvantaged groups. Although participation differed between groups, the levels of change for some sports and exercises were similar. For example, the proportion who adopted bowls, cycling and dancing was similar between occupational classes. These types of sport/exercise may be promising targets for strategies to promote PA in individuals from disadvantaged backgrounds.

We also found that specific sports and exercises in midlife were differentially associated with participating in sport/exercise and being active twenty years later, in old age. As well as golf, bowls also strongly predicted sport/exercise participation in old age. The associations for these sports are most likely due to the high levels of observed stability during the transition to old age. Most other sports and exercises were associated with a more moderately raised odds ratio for participating in sport/exercise in old age, and associations for cycling and cricket were weak. Similar patterns were observed between individual sports/exercises at baseline and total PA level twenty years later; however, only golf remained significant after adjusting for total PA at baseline. Despite low adherence to racquet sports, participation in midlife still strongly predicted sport/exercise participation and PA in old age. One possible explanation is that the skills acquired from racquet sports are more easily transferred to other types of sport/exercise. Indeed, we found that men who dropped out of racquet sports were likely to switch to another type of sport/exercise or were already engaged in other sports/exercises. The association between baseline racquet sport participation and physical activity level in old age was also attenuated when all sports/exercises and total physical activity were included in the model, suggesting that the relationship may also be explained, at least in part, by participation in other sports/exercises or physical activities. Indeed, more than half of racquet sports participants took part in multiple sports/exercises at baseline, which was high compared to other sports/exercises.

We also observed a dose-response relationship between the number of sports/exercises participated in during midlife and odds of still participating in sport/exercise and being active 20 years later, even after adjusting for total baseline PA levels. Plausibly, participating in multiple sports/exercises helps sustain fundamental movement skills that facilitate participation in a range of physical activities in old age. Previous studies in youth populations have shown that multiple sports participation is associated with greater neuromuscular control than single sport participation [11]. Increased neuromuscular control and physical function may be possible mechanisms explaining the relationship between multiple sports/exercise participation and PA maintenance into old age. Sampling of different types of sports and exercises should be encouraged, particularly in those who specialise in a specific sport/exercise, such as cycling.

Understanding the stability of specific sports/exercises may be particularly useful given the increasing evidence that the health benefits of sport/exercise might be specific to type. Previous findings from other British cohorts of older adults have identified specific sports/exercises, such as racquet sports, that are associated with lower mortality risks compared to other types of sport [2]. In the case of racquet sports, our findings suggest this may be due to multiple sport participation or high transferability of skills rather than long-term participation in racquet sports. In addition, previous controlled trials have found that 5–6 months of regular golf is associated with improved logical memory, adiposity and fitness compared to controls [24, 25]. Future studies should examine how changes and long-term participation in specific sports/exercises are associated with mortality and healthy ageing. Our findings highlight specific sports and exercises that could be targeted to promote lifelong participation and promote uptake in individuals who do not participate in midlife. Strategies to promote golf may be beneficial for promoting lifelong engagement but efforts to increase bowls may be particularly effective for engaging non-sports participants. Promoting multiple sport/exercise participation may be an effective strategy in single sport participants.

The key strength of this study is the long-term follow up of sport/exercise type participation, allowing the level of stability and change in participation to be determined. Although our sample is fairly large, only a small number of men took part in some specific sports and exercises. Consequently, the tracking of some sports and exercises could not be reliably estimated. Moreover, the questionnaire design meant that walking was reported elsewhere in the questionnaire under transport, so it may be possible that men did not report walking/hiking under the sport/exercise domain. In addition, we are unable to capture sport/exercise type participation during early life. Childhood is a crucial period for developing the fundamental movement skills that are necessary for lifelong PA, and there may be specific sports and exercises where early life participation is more crucial than others. Investigations using birth cohort studies with extended follow up into old age would help clarify how early life sport/exercise preferences influence later life engagement. Further, we know that men who dropped out of the study were less active and engaged in less sport/exercise. Subsequently, sport/exercise participation and stability may have been overestimated, although our conclusions were unchanged when men with only one measure of sport/exercise participation were included in random effects models. Further, there may have been some temporal shifts in the popularity of certain sports since our final follow up in 2000. For example, we know that dance participation has declined since the turn of the century [26], but golf, bowls, swimming and cycling remain among the most common sports in older British men [9]. Lastly, our sample is made up predominantly of white British men so these findings may not be generalizable to women, non-white ethnic groups and in different countries/cultures.

## Conclusion

The stability of sport/exercise participation from midlife to old age varies according to type. There are specific sports and exercises in midlife that are more likely to foster an active lifestyle into old age. However, encouraging participation in a diverse range of sports and exercises may be optimal for establishing a lifelong habit for sport and PA.

## Additional files

Additional file 1: Participation within sport and exercise groupings at baseline. (DOCX 18 kb)

Additional file 2: Participation and change in sport and exercise participation over 20 years of follow up in non-manual occupational classes, (*n* = 1657). (DOCX 16 kb)

Additional file 3: Participation and change in sport and exercise participation over 20 years of follow up in manual occupational classes, (*n* = 1727). (DOCX 16 kb)

## Abbreviations

BMI: Body mass index
BRHS: British regional heart study
ICCs: Intraclass correlation coefficients
PA: Physical activity
