# Supplementary material for: Tracking of sport and exercise types from midlife to old age: a 20-year cohort study of British men
Source: Eur Rev Aging Phys Act. 2018 Dec 7;15:16. doi: 10.1186/s11556-018-0205-y (PMC6284285; doi:10.1186/s11556-018-0205-y)
Supplement: Supplementary file 1 — Participation within sport and exercise groupings at baseline. (DOCX 18 kb) [file 11556_2018_205_MOESM1_ESM.docx]

Table S1. Participation within sport and exercise groupings at baseline

| **Sport group** | **Frequency** |
| --- | --- |
| **Bowling** |  |
| Bowls | 85% (63) |
| Curling | 4% (3) |
| Skittles | 11% (8) |
| **Dancing** |  |
| Ballroom | 50% (21) |
| Unspecified | 45% (19) |
| Sequence | 5% (2) |
| **Racquet sports** |  |
| Tennis | 24% (75) |
| Badminton | 35% (109) |
| Squash | 32% (101) |
| Table tennis | 9% (29) |
| **Surface water sports** |  |
| Sailing/boating | 79% (56) |
| Canoeing/Rowing | 16% (11) |
| Other water sports | 6% (4) |
| **Cycling** |  |
| Leisure Cycling | 10% (24) |
| Transport | 89% (219) |
| Exercise bike | 1% (3) |
| **Aerobics/fitness training^a^** |  |
| Keep fit classes | 23% (12) |
| Aerobics | 64% (33) |
| Cardiovascular machines | 14% (7) |
| **Gym/muscle strengthening** |  |
| **Walking/hiking** |  |
| **Swimming** |  |
| **Football** |  |
| **Golf** |  |
| **Running/jogging** |  |
| **Cricket** |  |
| **Rugby** |  |
| **Other (top 5 reported)** |  |
| Hunting/shooting | 13% (15) |
| Officiating | 12% (13) |
| Fishing | 15% (17) |
| Hockey | 9% (10) |
| Coaching | 9% (10) |

^a^ Prevalence from 20-year follow up
