# Supplementary material for: Tracking of sport and exercise types from midlife to old age: a 20-year cohort study of British men
Source: Eur Rev Aging Phys Act. 2018 Dec 7;15:16. doi: 10.1186/s11556-018-0205-y (PMC6284285; doi:10.1186/s11556-018-0205-y)
Supplement: Supplementary file 2 — Participation and change in sport and exercise participation over 20 years of follow up in non-manual occupational classes, (n = 1657). (DOCX 16 kb) [file 11556_2018_205_MOESM2_ESM.docx]

Table S2. Participation and change in sports participation over 20 years of follow up in non-manual occupational classes, (n=1657)

|  |  | |  | |  | |  | Change between baseline and 20-year follow up | | | |
| --- | --- | --- | --- | --- | --- | --- | --- | --- | --- | --- | --- |
| Sport/exercise type | Baseline | 12 year | | 16 year | | 20 year | | Participating at both | Adopters | Drop outs | Not participating at both |
|  | % (n) | | | | | | | | | | |
| Golf | 14.2 (235) | | 15.6 (259) | | 15.8 (262) | | 15.8 (262) | 9.3 (154) | 6.5 (108) | 4.9 (81) | 79.3 (1314) |
| Bowling | 1.9 (31) | | 8.0 (132) | | 9.1 (150) | | 9.7 (161) | 1.2 (20) | 8.5 (141) | 0.7 (11) | 89.6 (1485) |
| Dancing | 0.8 (13) | | 1.7 (28) | | 1.8 (29) | | 4.2 (69) | 0.1 (2) | 4.0 (67) | 0.7 (11) | 95.2 (1577) |
| Racquet sports | 14.2 (235) | | 5.9 (98) | | 3.5 (58) | | 3.4 (57) | 2.3 (38) | 1.2 (19) | 11.9 (197) | 84.7 (1403) |
| Swimming | 8.8 (145) | | 12.1 (200) | | 11.7 (193) | | 11.5 (190) | 2.6 (43) | 8.9 (147) | 6.2 (102) | 82.4 (1365) |
| Cycling (any purpose) | 5.3 (87) | | 8.6 (142) | | 8.0 (133) | | 5.6 (93) | 0.8 (13) | 4.8 (80) | 4.5 (74) | 89.9 (1490) |
| Surface water sports | 3.1 (52) | | 1.4 (23) | | 1.0 (16) | | 0.6 (10) | 0.3 (5) | 0.3 (5) | 2.8 (47) | 96.6 (1600) |
| Aerobics/fitness training | 0.2 (4) | | 1.2 (20) | | 1.3 (21) | | 1.9 (32) | 0.1 (1) | 1.9 (31) | 0.2 (3) | 97.9 (1622) |
| Gym/muscle strengthening | 1.7 (28) | | 1.9 (31) | | 3.0 (50) | | 2.9 (48) | 0.2 (3) | 2.7 (45) | 1.5 (25) | 95.6 (1584) |
| Football | 0.8 (13) | | 0.1 (1) | | 0.2 (4) | | 0.1 (2) | -- | 0.1 (2) | 0.8 (13) | 99.1 (1642) |
| Rugby | 0.1 (2) | | 0.0 (0) | | 0.0 (0) | | 0.0 (0) | -- | -- | 0.1 (2) | 99.9 (1655) |
| Running/jogging | 3.2 (53) | | 1.8 (29) | | 1.4 (23) | | 1.3 (21) | 0.5 (8) | 0.8 (13) | 2.7 (45) | 96.1 (1591) |
| Cricket | 2.2 (36) | | 0.2 (3) | | 0.2 (4) | | 0.2 (3) | 0.1 (2) | 0.1 (1) | 2.1 (34) | 97.8 (1620) |
| Walking/hiking | 2.2 (37) | | 8.0 (132) | | 9.7 (161) | | 10.4 (173) | 1.0 (16) | 9.5 (157) | 1.3 (21) | 88.3 (1463) |
| Other | 3.7 (62) | | 2.6 (43) | | 2.4 (40) | | 1.5 (25) | 0.3 (5) | 1.2 (20) | 3.4 (57) | 95.1 (1575) |
