# Supplementary material for: Tracking of sport and exercise types from midlife to old age: a 20-year cohort study of British men
Source: Eur Rev Aging Phys Act. 2018 Dec 7;15:16. doi: 10.1186/s11556-018-0205-y (PMC6284285; doi:10.1186/s11556-018-0205-y)
Supplement: Supplementary file 3 — Participation and change in sport and exercise participation over 20 years of follow up in manual occupational classes, (n = 1727). (DOCX 16 kb) [file 11556_2018_205_MOESM3_ESM.docx]

Table S3. Participation and change in sports participation over 20 years of follow up in manual occupational classes, (n=1727)

|  |  | |  | |  | |  | Change between baseline and 20-year follow up | | | |
| --- | --- | --- | --- | --- | --- | --- | --- | --- | --- | --- | --- |
| Sport/exercise type | Baseline | 12 year | | 16 year | | 20 year | | Participating at both | Adopters | Drop outs | Not participating at both |
|  | % (n) | | | | | | | | | | |
| Golf | 8.8 (152) | | 8.2 (141) | | 7.8 (135) | | 7.8 (134) | 4.6 (80) | 3.1 (54) | 4.2 (72) | 88.1 (1521) |
| Bowling | 2.5 (43) | | 8.9 (153) | | 10.0 (172) | | 9.3 (160) | 1.8 (31) | 7.5 (129) | 0.7 (12) | 90.0 (1555) |
| Dancing | 1.7 (29) | | 1.8 (31) | | 2.3 (39) | | 6.0 (103) | 1.0 (18) | 4.9 (85) | 0.6 (11) | 93.4 (1613) |
| Racquet sports | 4.6 (79) | | 1.5 (25) | | 0.9 (16) | | 0.9 (16) | 0.6 (10) | 0.4 (6) | 4.0 (69) | 95.1 (1642) |
| Swimming | 5.2 (90) | | 7.2 (124) | | 6.5 (112) | | 7.1 (122) | 1.1 (19) | 6.0 (103) | 4.1 (71) | 88.8 (1534) |
| Cycling (any purpose) | 9.2 (159) | | 9.2 (158) | | 8.3 (143) | | 6.5 (113) | 1.9 (33) | 4.6 (80) | 7.3 (126) | 86.2 (1488) |
| Surface water sports | 1.1 (19) | | 0.5 (9) | | 0.5 (9) | | 0.3 (5) | 0.1 (1) | 0.2 (4) | 1.0 (18) | 98.7 (1704) |
| Aerobics/fitness training | 0.1 (1) | | 0.2 (4) | | 0.4 (6) | | 1.2 (20) | -- | 1.2 (20) | 0.1 (1) | 98.8 (1706) |
| Gym/muscle strengthening | 0.6 (10) | | 1.4 (24) | | 1.5 (25) | | 1.0 (17) | 0.1 (1) | 0.9 (16) | 0.5 (9) | 98.5 (1701) |
| Football | 0.7 (12) | | 0.2 (3) | | 0.1 (2) | | 0.0 (0) | -- | -- | 0.7 (12) | 99.3 (1715) |
| Rugby | 0.2 (4) | | 0.0 (0) | | 0.0 (0) | | 0.0 (0) | -- | -- | 0.2 (4) | 99.8 (1723) |
| Running/jogging | 1.4 (24) | | 0.8 (13) | | 0.7 (12) | | 0.9 (15) | 0.4 (6) | 0.5 (9) | 1.0 (18) | 98.1 (1694) |
| Cricket | 1.0 (18) | | 0.4 (6) | | 0.1 (2) | | 0.1 (1) | 0.1 (1) | -- | 1.0 (17) | 99.0 (1709) |
| Walking/hiking | 0.6 (10) | | 5.0 (87) | | 5.2 (90) | | 5.7 (99) | 0.2 (3) | 5.6 (96) | 0.4 (7) | 93.9 (1621) |
| Other | 3.0 (51) | | 1.8 (31) | | 1.5 (25) | | 1.9 (33) | 0.4 (7) | 1.7 (29) | 2.6 (45) | 95.3 (1646) |
